# Isolation, Identification, Activity Evaluation, and Mechanism of Action of Neuroprotective Peptides from Walnuts: A Review

**DOI:** 10.3390/nu15184085

**Published:** 2023-09-21

**Authors:** Li Zhang, Yu-Ying Bai, Zi-Shan Hong, Jing Xie, Yang Tian

**Affiliations:** 1 College of Food Science and Technology, Yunnan Agricultural University, Kunming 650201, China; zhangl25@126.com (L.Z.);; 2 Engineering Research Center of Development and Utilization of Food and Drug Homologous Resources, Ministry of Education, Yunnan Agricultural University, Kunming 650201, China; 3 Yunnan Provincial Key Laboratory of Precision Nutrition and Personalized Food Manufacturing, Yunnan Agricultural University, Kunming 650201, China; 4 Yunnan Provincial Engineering Research Center for Edible and Medicinal Homologous Functional Food, Yunnan Agricultural University, Kunming 650201, China; 5 School of Tea and Coffee, Puer University, Puer 665000, China

**Keywords:** walnut, neuroprotective peptides, isolation, identification, activity evaluation, action mechanism

## Abstract

As human life expectancy increases, the incidence of neurodegenerative diseases in older adults has increased in parallel. Walnuts contain bioactive peptides with demonstrated neuroprotective effects, making them a valuable addition to the diet. We here present a comprehensive review of the various methods used to prepare, isolate, purify, and identify the neuroprotective peptides found in walnuts. We further summarise the different approaches currently used to evaluate the activity of these peptides in experimental settings, highlighting their potential to reduce oxidative stress, neuroinflammation, and promote autophagy, as well as to regulate the gut microflora and balance the cholinergic system. Finally, we offer suggestions for future research concerning bioavailability and improving or masking the bitter taste and sensory properties of final products containing the identified walnut neuroprotective peptides to ensure successful adoption of these peptides as functional food ingredients for neurohealth promotion.

## 1. Introduction

Memory is an essential function in the life of animals, helping to record the information received and processed in the brain and representing the series of responses of the human brain to its experiences. Memory is measured by observing changes in an individual’s behaviour over a period of time after learning [1]. Memory loss is a type of memory disorder that can progress to cognitive impairment and, in severe cases, central nervous system disease. Neurodegenerative diseases are primarily characterised by memory or cognitive impairment as the main clinical symptom. For example, Alzheimer’s disease (AD) is a severe neurodegenerative disease that primarily affects the older population and is characterised by a gradual decline in cognitive functions such as memory, learning, judgement, orientation, language, and comprehension. This decline can last from 5 to 20 years and eventually leads to the loss of basic living skills, severely affecting the activities of daily living and social functioning of older people. The prevalence of dementia has been increasing in recent years, largely due to the ageing population; it is predicted that approximately 131 million people will be affected by the condition by 2050 [2]. The economic impact of dementia is significant, with the cost of care estimated at approximately $818 billion per year worldwide, representing a significant burden on society.

Despite extensive efforts, there are still no curative treatments for neurodegenerative diseases owing to the challenges of their late onset and the complex factors that affect brain function throughout the lifespan [3]. Cerebrolysin is a compound with both neurotrophic and neuroprotective effects. It is produced from porcine brain protein by protease hydrolysis and contains 75% free amino acids and 25% low-molecular-weight polypeptides (<10 kDa). Studies have shown that cerebrolysin has growth factor properties that promote neuronal survival and sprouting [4], along with the ability to induce membrane stabilisation and restore blood–brain barrier (BBB) function [5], making it a clinically effective treatment for AD. However, several studies have shown that using a mixture with the same amino acid composition and ratio as cerebrolysin did not result in the same degree of improvement [6]. This suggests that specific peptides found in natural cerebrolysin may play a more important role in improving memory. As a result, an increasing number of studies have focused on the neuroprotective effects of bioactive peptides, which are promising candidates for the development of neuroprotective agents [7].

Walnut (*Juglans regia* L.) has received substantial attention from manufacturers and researchers owing to its high nutraceutical value. Containing proteins, unsaturated fatty acids, and vitamins, walnut is considered a valuable biological resource in China for both medicinal and food purposes based on its high nutritional value [8]. For centuries, it has been believed that eating walnut kernels can improve brain function and intelligence because of their similarity to the shape of the human brain; walnuts have become known as a longevity fruit and education fruit. Recent studies have confirmed this belief, showing that walnut supplements can reduce the risk of neurodegenerative diseases and delay their onset and progression [9]. Walnuts are a rich source of polyunsaturated fatty acids such as linoleic acid (LA; C18:2ω-6) and alpha-linolenic acid (ALA; C18:3ω-3) [10]. Accumulating evidence shows that LA and ALA have neuroprotective properties. For instance, LA was reported to exhibit inhibitory activity against the cytotoxicity of amyloid-beta (Aβ) fibres and the uptake of cholesterol in a *Drosophila* model of AD [11]. In addition, long-term ALA supplementation activated extracellular signal-related kinase and protein kinase B (Akt) signalling in the hippocampus, resulting in changes in the hippocampal synaptic structure and number, ultimately having ameliorative effects on cognitive deficits in natural ageing [12]. The phenolic acids, flavonoids and phytosterols in walnuts also have antioxidant and free radical-scavenging properties, and studies have confirmed that these substances have good neurocognitive properties. Phenolic acids, especially ellagic acid (EA), gallic acid and chlorogenic acid are highly abundant in walnuts [13]. EA had a profound effect on protecting dopamine neurons in a rat model of lipopolysaccharide (LPS)-induced dopamine neuron damage, which was achieved by suppressing microglial nucleotide-binding domain-like receptor protein 3 (NLRP3) inflammasome signalling activation and reducing the expression of proinflammatory cytokines, including interleukin (IL)-1β, tumour necrosis factor (TNF)-α, and IL-18 [14]. Among the flavonoids, flavanols such as catechin, epicatechin gallate, epigallocatechin and epigallocatechin gallate may have beneficial effects on cognition, both acutely and after long-term administration. The role of dietary flavanols in improving cognition in neurodegenerative diseases has been reviewed previously [15]. Phytosterols have been shown to modulate molecular processes involved in AD, such as Aβ formation via β-amyloid precursor protein (APP) processing and by affecting the expression, activity, and availability of secretases [16]. These findings suggest that a nutritional diet rich in walnuts may be effective in improving or protecting against neurodegeneration.

Walnuts are also rich in health-promoting bioactive peptides with antioxidation [17], anti-hypertension [18], anti-cancer [19], anti-inflammation [20], anti-diabetic [21], and immunoregulatory [22] activities. In particular, several studies have shown that walnut peptides have neuroprotective properties and can improve cognitive function [23]. Therefore, bioactive peptides are being considered as agents for the prevention of cognitive decline and dementia owing to their potential neuroprotective and brain health benefits [24]. We here provide a comprehensive review of the methods used for the preparation, isolation, purification, and identification of the neuroprotective peptides found in walnuts. The different approaches used to evaluate the activity of these peptides in experimental settings are also summarised. In addition, the possible mechanisms of the neuroprotective effects of walnut peptides are highlighted. Finally, we provide an outlook on the current research status and future development trends of walnut bioactive peptides.

## 2. Preparation, Isolation, Purification, and Identification of Walnut Peptides

### 2.1. Preparation of Walnut Peptides

Currently, there are multiple techniques utilised in the production of bioactive peptides. These methods include natural extraction, chemical synthesis, the chemical hydrolysis of proteins (both acid and alkaline), enzymatic hydrolysis, genetic recombination, and microbial fermentation [25]. Although the natural extraction method is a straightforward process, it may not be able to produce specific peptides with unique therapeutic properties due to limitations in the availability of natural raw materials [25]. Chemical synthesis methods are most commonly used to produce pure peptides, which can result in high purity. However, these methods are expensive, result in numerous by-products, and involve environmentally unfriendly processes [26]. The enzymatic hydrolysis of proteins is a highly effective method for obtaining bioactive peptides owing to its advantages of high hydrolysis specificity, mild reaction conditions, high product purity, and low energy requirements [27]. The efficiency of enzymatic hydrolysis and the resulting biological activity of the product are dependent on various factors, including the protein source, enzyme specificity, degree of enzymatic hydrolysis, and hydrolysis conditions such as pH, time, temperature, the enzyme-to-substrate ratio, and the material-to-liquid ratio. Among these factors, the specificity of the enzyme is a critical determinant of the properties of the hydrolysates and the produced peptides [28]. Selecting the appropriate preparation method for different raw materials is crucial in obtaining peptides with a specific biological activity and chemical structure. Table 1 highlights the advantages and disadvantages of each method, emphasising the need for careful consideration in the production of peptides.

Walnut protein is considered a high-quality plant protein. To further enhance its processing characteristics and biological activity, it is commonly enzymatically hydrolysed to obtain walnut peptides. The peptides have been found to have numerous advantages over the protein itself. Walnut antioxidant peptides can be obtained by hydrolysing walnut protein using three different proteases (neutrase, alcalase, pepsin). The hydrolysate was evaluated for its antioxidant properties and active antioxidant peptide sequences were released during enzymatic hydrolysis [31]. The angiotensin-I-converting enzyme inhibitor peptide was ultimately obtained from the hydrolysed walnut protein isolate [32].

Various enzymes, including neutralase, alkaline protease, protamex, pepsin, trypsin, and viscozyme L, have been reported to produce peptides from walnut proteins with memory-improving properties. For instance, alkaline proteases can hydrolyse walnut protein to produce peptides (RLWPF, VLRLF) that inhibit oxidative stress and neuroinflammation, modulate the intestinal microflora and metabolites, and improve learning ability in mice [33]. Walnut-derived peptides (GGW, VYY, LLPF) can also be produced by plant hydrolase and pancreatin complexes to improve learning ability and alleviate memory loss in sleep-deprived rats [34]. To enhance the nutritional, biological, and economic value of bioactive peptides, the enzymatic hydrolysis process has been optimised with respect to various parameters, including enzyme type selection, enzyme temperature, enzyme-to-substrate ratio, pH, and time. For instance, Cheng et al. [35] utilised a single-factor experiment and orthogonal test to optimise the preparation process of walnut peptides. The best enzymatic preparation process was achieved with a pre-treatment citric acid concentration of 2%, the addition of 4% alkaline protease, a material-to-liquid ratio of 1:15, and an enzymatic digestion time of 4 h. These conditions resulted in a yield of 33.5% walnut peptides. They further assessed the impact of walnut peptides on memory function in mice through various tests, including water maze, skipping, and dark avoidance tests. The results indicated a positive correlation between walnut peptides and improved memory in mice, as evidenced by their enhanced performance in both active and passive avoidance tasks. Wang et al. [36] used trypsin and complex polysaccharide enzyme L to hydrolyse a walnut protein solution under the following hydrolysis conditions: enzyme/substrate ratio 1.0% (*W*/*W*), pH 7.0, hydrolysis at 55 °C for 12 h. They obtained 20 peptides with improved memory function and neuroprotective effects. Finally, to improve the efficiency of enzymatic hydrolysis, physical fields, such as ultrasound, microwave, and high pressure, were used to assist enzymatic hydrolysis in the production of walnut bioactive peptides.

### 2.2. Separation, Purification and Structural Identification of Walnut Peptides

To study the biological activity of peptides, it is crucial to obtain peptides through appropriate separation and purification methods. Various separation and purification methods are commonly used for this purpose, including ultrafiltration membrane separation, column chromatography, and high-performance liquid chromatography (HPLC). The chromatographic techniques include ion-exchange chromatography (IEC), gel permeation chromatography (GPC), macro-porous resin column chromatography, affinity chromatography (AC), HPLC, reversed-phase HPLC (RP-HPLC), and ultra-HPLC (UPLC). Table 2 provides a comprehensive list of these methods, which should be selected based on the properties of the samples to be separated, such as molecular weight, polarity, hydrophilicity, and hydrophobicity.

The isolation and purification of walnut peptides with neuroprotective effects can be achieved through a combination of methods such as membrane separation and chromatography techniques. Membrane separation is a process that preliminarily separates crude peptide mixtures based on their molecular weight. Peptides with a small molecular weight can be purified through column chromatography, while liquid chromatography (LC) separates and purifies a mixture by utilising the partition coefficient of the components between the stationary phase and the mobile phase. For example, Chen et al. [39] achieved the separation of a defatted walnut meal hydrolysate into three fractions with SP-825 macroporous adsorption (2.6 cm × 60 cm), and further purified the fraction with the highest activity using a medium-pressure LC preparation system (4.9 cm × 46 cm). Feng et al. [40] separated defatted walnut protein hydrolysates into five fractions using an ultrafiltration membrane based on the molecular weights of 10, 5, 3, and 1 kDa. The fraction with high activity, fraction V with a molecular weight <1 kDa, was further purified using a gel chromatography column (HiLoad™16/600 Superdex™ 30) to obtain fractions F1 and F2. Subsequently, F2, with high activity, was isolated using reversed-phase HPLC (Daisogel C18 column, 250 mm × 20 mm, 10 μm) resulting in the fractions P1-P5. Among these fractions, P2 and P4 were identified using ultra-HPLC-quadrupole (Q)-time-of -flight (TOF) mass spectrometry (MS), and six peptides were obtained, namely, TY, SGGY, SSE, AHSVGP, VRN, and NPAN.

After purification, the identification of the peptide’s structure is crucial to understanding its biological activity. The structural analysis of peptides involves methods such as *N*-terminal sequencing, nuclear magnetic resonance (NMR), and MS. Table 3 lists the advantages and disadvantages of the different methods used for the identification of peptides. MS has rapidly developed owing to its advantages of speed, high sensitivity, and the capability to detect *N*-terminally blocked peptides [41]. In particular, the advent of soft ionisation techniques in MS, such as matrix-assisted laser desorption ionisation (MALDI) and electrospray ionisation (ESI), has significantly advanced peptide identification analysis [42]. Compared to MALDI, the ESI ion source operates by spraying the sample under high pressure and utilising a Coulomb explosion to ionise molecules [43]. This technology has effectively bridged the gap between LC and MS detectors, such as ESI-MS [44] and ESI-MS/LC-MS [45], and is currently the preferred method for peptide analysis. To identify peptides, MALDI and ESI ion sources are commonly paired with high-resolution mass spectrometer detectors, such as orbitraps and TOF, including MALDI-MS/ESI-TOF-MS [46], and MALDI-TOF/TOF-MS [47]. Most of the walnut peptides with neuroprotective effects reported to date were identified by ESI-MS. For example, walnut-derived peptides (FY, SGFDAE) were identified by ultra-HPLC-ESI-Q-TOF-MS/MS as having neuroprotective capacity in scopolamine-induced zebrafish [36]. Walnut-derived peptides (TY, SGGY) play a protective role on neurotoxicity induced by D-galactose and aluminium chloride in mice, and these peptides were also identified by ultra-HPLC-ESI-QTOF-MS [22]. MS analysis commonly employs two methods: library searching and de novo sequencing. The library search method is mostly suitable for raw protein sequences that have already been reported, enabling the analysis of more than six peptides. The de novo method is used for the peptide analysis of less than six peptides and for proteins with no related sequence or complete sequence reports. This method involves manually matching *m*/*z* values and combining peptide fragment information for spectrum analysis. Sheng et al. [48] analysed the data obtained by the de novo sequencing software pNovo 3 and acquired a total of 2212 and 1536 peptides for the defatted walnut meal hydrolysates 1 and 2, respectively.

## 3. Assessment of Neuroprotective Effects of Walnut Peptides

Currently, basic research on the mechanisms underlying the efficacy of peptides to improve memory is mainly conducted using in vitro cell models, which are then evaluated and verified using in vivo animal models. The in vitro cell model is widely used to simulate neurophysiologic activities in the brain because of the advantages of a short experimental period, low cost, good repeatability, and high-throughput screening for target functions. This method is particularly useful for exploring molecular-level mechanisms such as changes occurring at the transcription and protein levels [51]. Animal models have been utilised to assess the efficacy of neuroprotective peptides. This is achieved by comparing the behavioural performance, physiological indicators, and brain tissue morphology of normal animals with those of the model animals [52].

### 3.1. In Vitro Assays

Memory impairment and cognitive decline are largely associated with damage to the hippocampal neuron cells. Therefore, it is more representative and feasible to use primary hippocampal neuron cells for experiments with neuroprotective peptides. However, primary neuron cells are susceptible to exogenous components and have a low survival rate, making their utilisation challenging [53]. Cell lines such as PC12 (rat pheochromocytoma cells), BV-2 (microglia), THP-1 (human acute monotypic leukaemia cells), and SH-SY5Y (human neuroblastoma cells), which are similar to neurons in morphology and physiological function, have been extensively used in the neurological research of walnut-derived neuroprotective peptides [54].

The construction of a cell injury model is crucial for studying the mechanism of specific neurodegenerative diseases. Researchers typically select appropriate inducers to construct cell models based on the target mechanism of neuroprotection. Commonly used chemical inducers include H_2_O_2_, Aβ_1–42_, Aβ_25–35_, glutamate (Glu), and LPS. Different inducers can lead to the different pathogenesis of neurodegenerative diseases. For instance, H_2_O_2_ is used for oxidative stress studies, Glu is used for neurotoxicity studies, and LPS is used for inflammatory response studies. It is important to note that using different inducers on the same cell type can result in varying levels of neurotoxicity and neurodegenerative disease pathogenesis. In the case of PC12 cells, H_2_O_2_ was utilised to induce cellular oxidative stress effects [39], whereas Glu treatment was used to assess neurotoxicity [34]. PC12, derived from adrenal medulla cells of a rat model of pheochromocytoma, is considered to be an ideal cell line for studying the physiology, pathology, and pharmacology of nerve cells. The protective effects of walnut peptides against neurodegenerative disease have been studied in various neural cell culture models, as described below.

PC12 cells are commonly used for in vitro studies of nervous system diseases [55]. Aβ_25–35_, Glu, and H_2_O_2_ are typically used to induce PC12 cells to establish cell models of neurodegenerative diseases. The induction of PC12 cells by modelling reagents mainly involves oxidative damage and apoptosis. For instance, Zhao et al. [56] conducted a study on the effect of three peptides derived from walnuts (TWLPLPR, YVLLPSPK, KVPPLLY) in alleviating oxidative stress in an in vitro model of PC12 cell neurotoxicity induced by Aβ_25–35_. They screened three walnut-derived peptides with higher inhibitory activity of reactive oxygen species (ROS) and apoptosis, along with improvements in adenosine 5-triphosphate (ATP) and glutathione peroxidase (GSH-Px), to explore the potential mechanism of the exogenous peptides in promoting autophagy and neuroprotection against Aβ-induced oxidative stress in PC12 cells [56].

The progression of neurodegenerative diseases often involves activation of the microglia. As the resident macrophage-like cells in the brain and spinal cord, microglia are the first line of defence against the entry of foreign particles or infectious agents. BV-2 cells were derived from Raf/Myc-immortalised murine neonatal microglia and are the most frequently used substitute for primary microglia [57]. LPS-treated microglia have been proposed as an in vitro model of microglia activation. Gao et al. [58] fully elucidated the protective mechanisms of the walnut peptide, WEKPPVSH, on LPS-stimulated BV-2 microglia; they investigated the effects of WEKPPVSH on ROS production, antioxidant enzymes [superoxide dismutase (SOD) and catalase (CAT)] activity, and the secretion of inflammatory factors [nitric oxide (NO), TNF-α, 1L-1β, 1L-6, inducible nitric oxide synthase (iNOS), and cyclooxygenase-2 (COX-2)] in LPS-induced BV-2 microglia.

The human neuroblastoma SH-SY5Y cell line, a subline of SK-N-SH cells, is an in vitro dopaminergic neuron model for neurological diseases, especially Parkinson’s disease [59]. The reagents commonly used to induce human neuroblastoma cells to establish neurodegenerative disease models include Aβ and H_2_O_2_, among others, and the induction time is most frequently 24 or 48 h. The main detection indicators include cell viability, apoptosis rate and related apoptosis protein expression, oxidative stress-related indicators, and the tau protein phosphorylation level. Feng et al., [60] investigated the neuroprotective effects of SGGY, a walnut-derived peptide, on H_2_O_2_-stimulated oxidative stress in SH-SY5Y cells and explored the underlying mechanisms. The results showed that exposure to H_2_O_2_ led to a significant reduction in cell viability and an increase in lactate dehydrogenase (LDH) leakage in the culture medium, indicating damage to the cell membrane, ultimately resulting in cell death. The study also found that exposure to H_2_O_2_ led to a significant increase in intracellular ROS levels and a loss of mitochondrial membrane potential (MMP). However, treatment with SGGY notably reversed the intracellular ROS levels, prevented the decline of MMP, ameliorated the leakage of LDH, and increased cell viability. Moreover, this study suggested that the JNK, p38, and Nrf2 signalling pathways may be involved in the observed effects of SGGY [60].

### 3.2. In Vivo Assays

Neuroprotective peptides are frequently evaluated using animal models. Common experimental animal models of memory and cognitive impairment include ageing models, transgenic models, and the injection of exogenous harmful substances [61].

Animal experiments on ageing models typically utilise natural ageing models, rapid ageing models, and galactosamine (D-gal) models. The natural ageing model is constructed by prolonging feeding times, providing an advantage of similarity to the human ageing process and memory decline in old age. However, a disadvantage is the difficulty in forming Aβ senile plaques and neurofibrillary tangles due to pathological changes in the brain. The long breeding time of this model leads to high mortality and high costs, and naturally ageing animals cannot be raised in large quantities [62]. Animal experiments on rapid ageing models are primarily based on natural variations in animals. These experiments offer clear advantages in terms of changes in behavioural cognition, the abnormal metabolism of neurotransmitters and free radicals, as well as Aβ deposition. However, the costs associated with such experiments are often deemed too high [63]. The D-gal animal model is primarily used to induce metabolic disorders through daily intraperitoneal injection of amino-galactose, resulting in animal ageing. The D-gal animal model is commonly used to evaluate neuroprotective peptides in vivo because of the advantages of a controllable modelling time, stable results, and low cost [64]. For instance, Dang et al. [65] induced memory impairments in mice using D-gal and then investigated the protective effect of the walnut-derived peptide, TW7, in the Morris water maze test. The results demonstrated that TW7 improved the learning ability and memory of the mice with cognitive impairments. Transmission electron microscopy further showed that BBB integrity in the hippocampus was restored. Additionally, immunofluorescence analysis and Western blotting indicated that the protection of BBB integrity was linked to increased expression levels of tight junction proteins.

The transgenic animal models used in memory impairment experiments are primarily genetically constructed models. Commonly used models include single-mutation animal models, such as the APP transgenic model, PS1 transgenic model, and tau transgenic model, as well as compound transgenic models such as the APP/PS1 double transgenic model and the APP/PSI/tau triple transgenic model. Transgenic models are constructed to have a clear aetiology and to study mechanisms. These models are able to fully simulate pathological changes, such as neuronal damage, synaptic loss, and Aβ deposition, as well as age-related cognitive and behavioural deficits [66]. For example, Wang et al. [67] used the classic APP/PS1 mouse model to validate that the walnut-derived peptide, PW5, exerts its effects on cognitive improvement through reducing the accumulation of Aβ plaques.

To induce memory loss and cognitive decline, researchers inject exogenous toxic substances into the animal’s brain or intraperitoneal cavity. These substances can include colchicine, Aβ_1–42_, sodium azide, and scopolamine. However, it is important to note that administering too small a dosage may result in a failed model, while administering too large a dosage may lead to the animal’s death [68]. Scopolamine is commonly used in experiments to induce cognitive disorders owing to its ability to easily cross the BBB. In the case of AD, scopolamine has been shown to cause dysfunction in the cholinergic system and increase the deposition of Aβ. Geng et al. [69] investigated the memory improvement effects of a walnut-derived peptide, WNP-10. For assessment, they utilised the Morris water maze test, ultrastructural observation of the mitochondrial morphology in hippocampus tissues, hematoxylin and eosin-staining for the pathological morphology of the hippocampus, and Nissl staining to evaluate the cellular structure of the hippocampus, demonstrating that WNP-10 significantly enhanced the learning and memory capabilities of scopolamine-injured mice.

In addition, animal models of sleep deprivation have been utilised to study memory and cognitive function. Sleep deprivation is a significant contributor to the neurodegenerative disorders linked to memory deficits. The impairment of short-term and long-term memory, which rely on the hippocampus, is one of the consequences of sleep deprivation [70]. Neurodegenerative diseases can cause significant impairment of the sleep cycle in 40–90% of patients due to the degeneration of hypothalamic and brainstem neurons. Recent findings suggest that chronic sleep deprivation can also lead to the activation of immune inflammatory responses in brain regions and pathological changes similar to those observed in neurodegenerative diseases. Interestingly, these changes have been observed in both healthy individuals and in those with neurodegenerative diseases [71]. This model has the advantage of simulating the neurological disorders related to memory loss caused by sleep deprivation in humans. Wang et al. [34] determined the neuroprotective effects of walnut protein hydrolysate (WPH) and especially its low-molecular-weight fraction (WPHL < 3 KDa) against memory deficits induced by sleep deprivation in rats. The improvement in learning and memory performance might be related to its antioxidative activity.

In summary, the evaluation of substances with memory-improving activity has become a common practice through the combined use of chemical analysis, cell models, and animal models.

**Figure 1:**
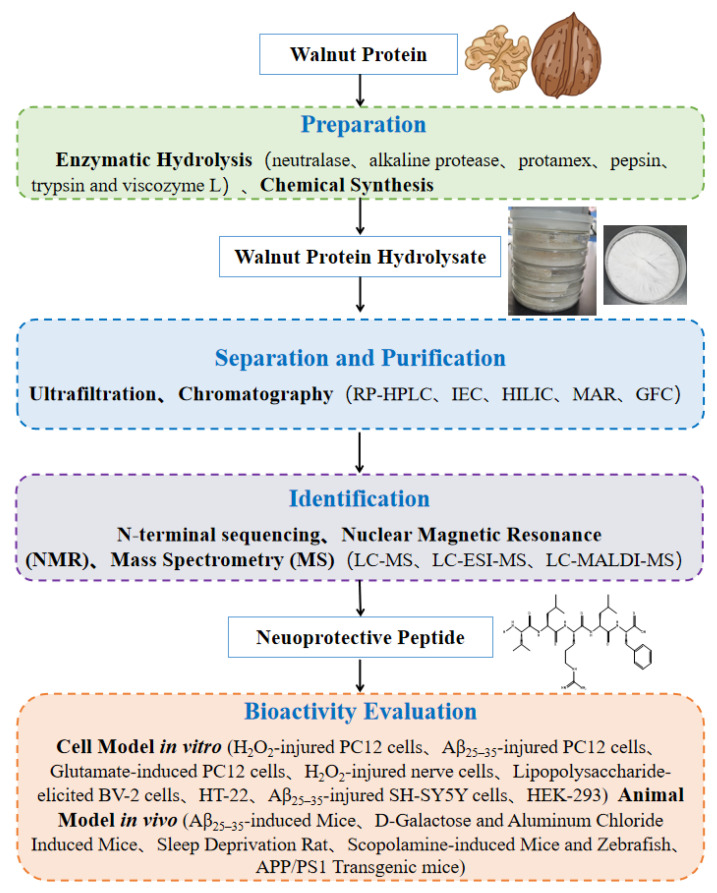
Procedures for extraction, purification, identification, and bioactivity evaluation of walnut neuroprotective peptides.

Procedures for extraction, purification, identification, and bioactivity evaluation of walnut neuroprotective peptides were summaried in Figure 1.

## 4. Mechanisms of the Neuroprotective Effects of Walnut Peptides

**Figure 2:**
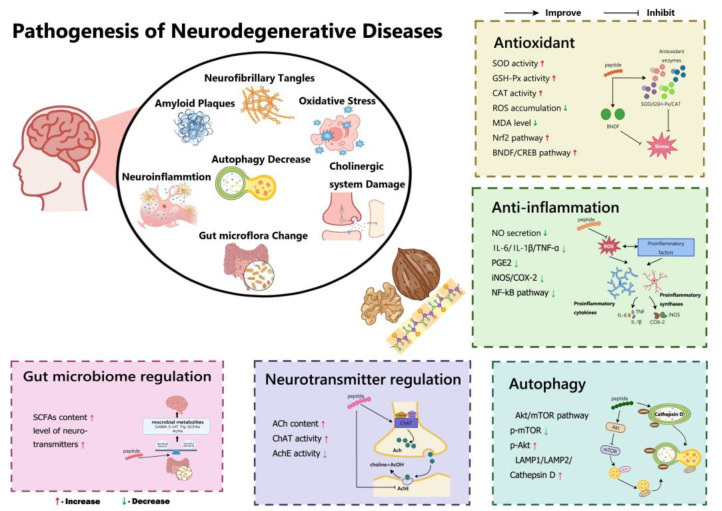
The pathogenesis of neurodegenerative diseases and the regulatory mechanisms of walnut neuroprotective peptides.

At this stage of research, the main causes of neurodegenerative diseases are considered to be either neuronal loss due to apoptosis or progressive degenerative changes in the structure and function of neuronal cells without a significant decrease in the number of neuronal cells [72]. Neurodegenerative disorders and diseases are marked by a variety of pathologies, including oxidative damage, neuroinflammation, mitochondrial dysfunction, impaired neurotrophic signalling, tau protein phosphorylation, the formation of neurofibrillary tangles, extracellular deposition of amyloid plaques, and disorder of neurotransmitters, all of which ultimately lead to neuronal death [73,74]. Recent studies highlight that walnut peptides exhibit neuroprotective properties, which have been confirmed through various means such as their antioxidant and anti-inflammatory effects, ability to enhance autophagy, regulation of the cholinergic system, and improvement of the intestinal microflora (Figure 2).

### 4.1. Antioxidant Effects

Numerous studies have demonstrated the significant involvement of oxidative stress in the functional impairment of mitochondria [75], apoptosis [76], endoplasmic reticulum stress [77], neurotransmitter release, and transport. This highlights the crucial role that oxidative stress plays in the development of neurodegenerative diseases [78]. According to the free radical theory, an overabundance of free radicals can cause neuronal destruction through apoptosis and can disrupt the body’s natural antioxidant defence system. This can result in damage to various biological and pathological processes, including ageing and memory impairment [79]. Oxidative stress occurs when there is an imbalance between free radicals and antioxidant defences in the body. ROS are harmful and can react with lipids, proteins, and nucleic acids in cells, disrupting their function. This in turn affects membrane properties, including fluidity, enzyme activity, ion transport, and protein cross-linking. Over time, oxidative stress can cause significant damage and even cell death [80]. The brain’s high rate of oxygen consumption makes it more vulnerable to oxidative stress than any other organ. The brain utilises 20% of the body’s total oxygen intake and contains unsaturated fatty acids while having limited antioxidant enzymes [81]. This puts neurons at a higher risk of oxidative stress, which can cause direct damage to the central nervous system [82]. Therefore, it may be possible to alleviate neurodegenerative diseases by protecting the brain from oxidative stress-induced damage.

Numerous studies have demonstrated the significant impact of walnut peptides on antioxidant activity. For example, Chen et al. [39] found that walnut protein hydrolysate exhibited protective properties against H_2_O_2_-induced oxidative stress by promoting cell proliferation and inhibiting apoptosis in PC12 cells. The oxygen radical absorbance capacity (ORAC) value of the peptide, WSREEQEREE, from walnut was 2.95 μmol TE/μmol, which was 4.4 times higher than that of glutathione (0.69 μmol TE/μmol). Additionally, the ORAC value of ADIYTEEAGR was 2.38 μmol TE/μmol, indicating that it had better antioxidant activity in vitro, which was 3.5 times higher than that of glutathione (GSH). In mice, walnut protein hydrolysate has been shown to potentially improve memory impairment caused by D-gal. This effect may be attributed to the hydrolysate’s strong antioxidant properties. Sheng et al. [83] identified that VEGNLQVLRPR and HNLDTQTESDV from walnut protein hydrolysates caused a decrease in intracellular ROS levels. Wang et al. [34] discovered that the walnut-derived peptides, GGW, VYY, and LLPF, had a significant neuroprotective effect on PC12 cells treated with glutamate by reducing intracellular ROS production. GGW and VYY increased the activities of antioxidant enzymes, such as SOD and GSH-px, while LLPF inhibited Ca^2+^ influx and matrix metalloproteinase inactivation. These findings suggest that the ability of walnut peptides to enhance learning and memory is associated with their antioxidation effects. Feng et al. [84] examined the potential neuroprotective effects of the walnut-derived peptide, SGGY, on oxidative stress induced by H_2_O_2_ in SH-SY5Y cells. The findings indicated that SGGY was successful in reducing intracellular ROS levels and altering mitochondrial MMP, which led to the inhibition of cell apoptosis and an improvement in cell viability; however, these results were based on the 3-(4,5-dimethylthiazol-2-yl)-2,5-diphenyltetrazolium bromide (MTT) assay, which is not considered to be very reliable. Nevertheless, these results suggest that SGGY may have therapeutic potential for the treatment of oxidative stress-related neurodegenerative diseases. SGGY significantly restored the activities of antioxidant enzymes and decreased the content of malondialdehyde. The mechanism may be related to the mitogen-activated protein kinase (MAPK) and Nrf2 signalling pathways.

### 4.2. Anti-Inflammatory Effects

Chronic neuroinflammation is a defining characteristic of neurodegenerative diseases. Glial cells, which are resident immune cells of the central nervous system, play a critical role in detecting extracellular stimuli and initiating neuroinflammatory responses. When activated, these cells produce pro-inflammatory cytokines, such as TNFα, IL-6, IL-2 and IL-1β, as well as reactive oxygen and nitrogen species, ultimately leading to inflammation-induced neuronal cell death [80]. Moreover, the intracellular and intercellular accumulation of abnormally aggregated proteins, such as Aβ in damaged neural tissue, leads to persistent neuronal inflammation. Neuroinflammatory processes and oxidative stress are interdependent and cause a cascade effect. Oxidative stress induces pro-inflammatory effects by activating multiple pathways, such as the NF-κB pathway, and increasing pro-inflammatory gene expression, leading to neuroinflammation [85]. There is increasing evidence that neuroinflammation is an early event in the pathogenesis of neurodegeneration, which is often associated with oxidative damage [80]. To assess neuroinflammation and its associated inflammatory responses, one can evaluate the activation of resident central nervous system (CNS) immune cells (including microglia), BBB permeability, cytokine production, demyelination, cell death, tau protein misfolding, immune cell infiltration of the central nervous system, and morphological changes and tissue destruction [86]. Hence, antineuroinflammation could be an effective strategy for the prevention or treatment of neurodegenerative diseases.

Research conducted both in vivo and in vitro has demonstrated that bioactive peptides exhibit neuroimmunotrophic activity, which in turn reduces inflammation and expedites neuronal death in pathological scenarios [87]. Recent studies have identified a growing number of bioactive peptides in the protein component of walnuts [88]. These peptides have been found to effectively reduce the neuroinflammation caused by excessive inflammatory cytokines in neurodegenerative diseases. Wang et al. [89] found that the walnut peptides, LPF, GVYY, and APTLW, were effective in improving memory damage caused by LPS. This was achieved through the modulation of inflammatory responses and oxidative stress in the brain. The researchers further demonstrated the peptides’ strong anti-inflammatory effects in downregulating iNOS, COX2, and p-IkB/IkB expression; inhibiting the overproduction of pro-inflammatory mediators, including NO and prostaglandin E2; and reducing the expression levels of TNF-α, IL-1β, and IL-6 in LPS-stimulated BV-2 microglia. Gao et al. [58] investigated the antioxidant and anti-inflammatory protective effects of the walnut peptide, WEKPPVSH, on LPS-induced BV-2 microglia and its possible mechanisms. The study demonstrated that WEKPPVSH had a dose-dependent effect on reducing the production of NO and ROS, while also increasing the activities of SOD and peroxidase. Additionally, this peptide significantly reduced the secretion of TNF-α, IL-1β, and IL-6. The possible mechanism underlying these effects was that WEKPPVSH protected LPS-stimulated BV-2 microglia from oxidative stress and inflammation by enhancing the Nrf2/HO-1 signalling pathway and blocking the NF-κB/p38 MAPK signalling pathway. Another study found that the peptide, EVSGPGLSPN, derived from walnuts can reduce the levels of the inflammatory factors IL-1β and TNF-α in PC12 cells when exposed to H_2_O_2_. This indicates that walnut peptides may have the ability to inhibit the inflammatory cascade in the brain, potentially preventing neurodegeneration associated with ageing [90].

### 4.3. Autophagy Induction

Autophagy is a vital and widespread process in eukaryotic cells, playing a crucial role in neuronal function through the removal of misfolded proteins and organelles caused by lysosomes. The resulting degradation products are then utilised to provide energy and rebuild the cell structure. However, autophagic activity declines with age and contributes to the development and progression of AD pathology through interactions with Aβ and tau. In particular, defects in autophagy lead to the accumulation of immature autophagic vesicles filled with Aβ peptides. These autophagic vacuoles not only provide a convenient site for Aβ aggregation but also provide a new pathway for Aβ production [91,92]. Defects in autophagy also affect tau degradation and phosphorylation status. In recent years, autophagy has been regarded as a novel neuroprotective strategy, given its pivotal role in the pathogenesis of AD.

Zhao et al. [56] extracted three novel peptides, TWLPLPR, YVLLPSPK, and KVPPLLY, from walnuts. These peptides were found to regulate the Akt/mammalian target of rapamycin (mTOR) signalling pathway through p-Akt (Ser473) and p-mTOR (S2481), thereby promoting autophagy by increasing LC3-II/LC3-I and Beclin-1 levels, while decreasing p62 expression. This study also showed that the peptides were able to increase the levels of LAMP1, LAMP2, and histone D, resulting in the promotion of fusion with lysosomes to form autolysosomes, which in turn accelerated the clearance of ROS. Based on these findings, it can be concluded that nucleopeptides play a role in regulating oxidative stress by promoting Aβ_25–35_-induced autophagy in PC12 cells. In addition, mitochondrial dysfunction is closely associated with neurodegenerative diseases. Perez Ortiz et al. [93] observed alterations in the mitochondria of patients with AD. Specifically, they found that the accumulation of Aβ proteins, a hallmark of AD pathology, can result in mitochondrial dysfunction. Additionally, mitochondria are highly susceptible to oxidative stress and the process of mitochondrial autophagy plays a critical protective role in the development of neurological diseases. Yang et al. [94] discovered that a peptide derived from walnuts, known as TW-7, exhibits antioxidant activity. Furthermore, they found that TW-7 can restore both the morphology and function of mitochondria through the process of JNK-regulated PINK1-mediated mitochondrial autophagy. This ultimately leads to a delay in neuronal damage caused by oxidative stress, thus demonstrating the neuroprotective effect of mitochondrial autophagy against ROS-induced neurodegenerative diseases. Zhao et al. [95] found that the walnut-derived peptide, YVLLPSPK, improved learning and memory in scopolamine-induced cognitively impaired mice. This effect was achieved through the activation of PINK1-mediated mitochondrial autophagy within the NRF2/KEAP1/HO-1 pathway.

### 4.4. Regulation of the Cholinergic System

The cholinergic hypothesis states that the degeneration of cholinergic neurons and the associated loss of cholinergic neurotransmission (e.g., acetylcholine) in the cerebral cortex are responsible for the deterioration of cognitive function observed in the brains of patients with AD [96]. The brains of patients with AD exhibit reduced activity of acetylcholinesterase (AChE) and acetylcholinesterase (ChAT), as well as a significant deficiency in acetylcholine. Experiments involving the cholinergic system and memory have shown that administering cholinergic antagonists to mice leads to reduced discrimination and impaired memory. This results in cognitive impairment, which can be alleviated by administering cholinergic agonists [97], providing further confirmation of the association between an impaired cholinergic system and memory impairment. Cholinergic impairment can contribute to the development of AD, both independently and in conjunction with other pathological factors. Research conducted on animals has shown that a cholinergic imbalance can accelerate the deposition of Aβ [98]. Additionally, abnormal cholinergic changes in the central nervous system can lead to abnormal tau protein phosphorylation, neuronal inflammation, apoptosis, and imbalances in the neurotransmitter and neurohormonal systems. These pathological phenomena are only a few examples of the potential impacts resulting from cholinergic impairment [99]. Therefore, modulation of cholinergic function to restore acetylcholine levels in the brain has been identified as a viable strategy for treating AD.

Numerous studies have reported the neuroprotective effect of walnut peptides through the modulation of choline function. For instance, the hydrolysate of walnut protein can reduce the levels of ChAT and acetylcholine in the brain tissue of mouse models induced by D-gal and AlCl_3_. This treatment also significantly increases the activity of the AChE enzyme and reverses cholinergic dysfunction [22]. Wang et al. [33] found that administering walnut protein hydrolysate orally to mice significantly improved their behavioural performance. This also helped in restoring the cholinergic system disorder and oxidative stress in the mouse brain. Walnut protein hydrolysate was also found to upregulate the antioxidant defence-related protein Nrf2 and the expression of neurotrophin-related proteins (BDNF and CREB). Docking with AChE and Keap1 predicted 20 peptides with relatively high abundance and PeptideRanker scores. Among these, FY and SGFDAE had the highest binding affinity of −9.8 and −8.0 kcal/mol, respectively, and were considered as promising AChE and Keap1 inhibitors. These inhibitors were further verified to have neuroprotective abilities in scopolamine-induced zebrafish.

### 4.5. Improvement of the Gut Microbiota

The gut–brain axis is a bidirectional communication network between the gut and the brain. Gut microflora can influence brain function and behaviour through the gut–brain axis [100]. Research indicates a correlation between the development of AD and intestinal disorders. Studies have shown that patients with AD have a significant change in their intestinal flora composition compared to that of healthy individuals [101]. The intestinal flora can impact the central nervous system in three ways. First, the bacteria in the intestines can produce and release compounds that loosen the tightness of the intestinal barrier. This accelerates the penetration of intestinal microorganisms into the body, leading to a systemic inflammatory response. Prolonged chronic inflammation can cause a disorder in the BBB, which in turn induces a central inflammatory response, ultimately triggering AD [102]. Second, intestinal microorganisms can produce various small-molecule metabolites, including monoamines, amino acids, and short-chain fatty acids. These metabolites can be transported to different parts of the body, including the central nervous system, through the lymphatic and blood systems. As a result, they can affect normal physiological activities. Another study found that a significant change in the intestinal flora of AD mice led to increased levels of phenylalanine and isoleucine in the blood. This increase induced peripheral inflammation, which promoted the infiltration of peripheral immune cells into the brain, causing central inflammation and ultimately triggering AD [103]. Finally, the gut microbiota can play a role in regulating the central nervous system by producing substances that have neurotransmitter activity, such as serotonin, epinephrine, dopamine, acetylcholine, and gamma-aminobutyric acid.

Emerging data have indicated that peptides, especially walnut peptides, could exert cognitive-improving effects by modulating the gut microbiota community and diversity [104]. For example, studies have demonstrated that peptide sequences derived from walnuts, specifically RLWPF and VLRLF, can enhance the effects of D-gal-induced cognitive impairment by acting on the microbial–gut–brain axis. Treatment with RLWPF and VLRLF significantly improved the spatial learning and memory impairment caused by D-gal. According to 16S rRNA sequencing analysis, the treatment of RLWPF and VLRLF was found to improve cognitive impairment by altering the composition of gut microbiota and reducing the abundance of harmful bacteria such as the *Firmicutes*-to-*Bacteroidetes* ratio, *Helicobacter*, *Allobaculum*, *Alistipes*, *Mucispirillum*, and *Odoribacter*. Along with this regulation, both RLWPF and VLRLF have their own distinct markers and regulatory flora [33]. In addition, walnut protein hydrolysate had a significant impact on reducing Aβ plaque accumulation in the brain of a APP/PS1 transgenic mouse model. The peptide, Pro-Pro-Lys-Asn-Trp (PW5), identified from walnut protein hydrolysate also showed a similar inhibitory effect. Additionally, the study confirmed that the restoration of gut microbiota in APP/PS1 transgenic mice treated with PW5 resulted in a decrease in *Firmicutes* and an increase in *Proteobacteria* [67].

Table 4 summarises the production and regulatory mechanisms of walnut neuroprotective peptides highlighted in recent research.

## 5. Conclusions and Future Perspectives

The prevalence of neurodegenerative diseases has risen to alarming levels due to environmental pollution and the consumption of highly processed foods [100]. As a result, the prevention and treatment of such diseases have become crucial. One promising approach to treating cognitive impairment is through the use of bioactive peptides. There is a rising interest in finding neuroprotective peptides or protein hydrolysates that are natural, potent, and affordable. Researchers are also trying to comprehend the mechanisms behind the benefits of these peptides on brain health and function, especially in the cognitive disorders that accompany neurodegenerative diseases [54]. Numerous studies have confirmed that walnut peptides are a beneficial food ingredient for cognitive impairment. This article provides an overview of the potential neuroprotective effects of walnut peptides, summarising the various methods used in the preparation, isolation, purification, identification, biological activity evaluation, and elucidation of the neuroprotective mechanism of walnut-derived neuroprotective peptides. The mechanisms discussed include reducing oxidative stress and neuroinflammation, increasing autophagy, enhancing cholinergic activity, and regulating the gut microbiota. However, a prerequisite for bioactive peptides to exert physiological effects in vivo is that they survive in the gastrointestinal tract during digestion and are effectively absorbed to reach their target sites. Accordingly, comprehensive consideration of the bioavailability and bioactivity of food-derived peptides is necessary [49]. According to Gallego et al. [109], the peptide, SNAAC, exhibited a significant decrease in antioxidant activity as a result of the removal of the terminal Cys residue during simulated gastrointestinal digestion. Similarly, the antioxidant peptide, AEEEYPDL, was broken down into smaller peptides, AEEEY and PDL, after simulated gastrointestinal digestion, leading to the loss of its antioxidant activity. Zhao et al. [110] investigated the impact of in vitro-simulated gastrointestinal digestion on the biological activity of the neuroprotective octapeptide, WCPFSRSF. They were able to identify eight novel peptides, namely, WCP, WCPF, WCPFS, PF, PFS, SR, SRS, and WCPFSRS, from the final digestive production. It is worth noting that SR and WCPF demonstrated neuroprotective capabilities against excitotoxicity in cells. Wang et al. [105] discovered that the walnut neuroprotective peptides, WSREEQEREE and ADIYTEEAGR, were completely degraded during in vitro digestion. This finding suggests that these peptides are susceptible to enzymes in the gastrointestinal tract. Building upon previous research on digestive stabilisation, the researchers proposed that encapsulating WSREEQEREE and ADIYTEEAGR is necessary to preserve their integrity during digestion and absorption, thereby ensuring their neuroprotective efficacy. Therefore, future research should fully consider the bioavailability of walnut neuroprotective peptides.

Moreover, it is important to note that the data reviewed herein are not based on clinical trials and the level of evidence obtained by conducting relevant clinical trials would further enhance our understanding of the neuroprotective properties of walnut peptides and establish a clear structure–function relationship in the prevention and treatment of cognitive dysfunction. Nevertheless, based on current evidence, the potential of walnut peptides to act as neuroprotective agents has been confirmed, offering a promising candidate for use in the development of functional foods. However, it is important to note that bioactive hydrolysates and peptides often have a bitter taste, which may negatively impact consumer acceptance. As such, future attention should be paid to addressing the negative sensory properties of end products containing walnut peptides, as consumers are often unwilling to sacrifice taste for potential health benefits [111]. Numerous studies have highlighted the importance of taste expectations and experiences in determining the selection of functional foods [112]. However, no research has yet been conducted to identify and describe the particular bitter peptides present in walnuts and their by-product proteins. Additionally, various methods have been employed to reduce bitterness in other protein hydrolysates, such as the elimination of the hydrophobic peptides that are known to contribute to bitterness [113]. However, the hydrophobic amino acids that contribute to the bioactivity of peptides may be essential; therefore, removing them may affect the bioactivity of the hydrolysate. Further research is needed to understand the relationship between active and bitter peptides of walnut. It is recommended that the functional characteristics of isolated walnut peptides be studied to reduce the production of bitter peptides. Developing effective bitterness-inhibiting or masking agents will aid in expanding the application of bioactive peptides and hydrolysates as functional food ingredients.

## Figures and Tables

**Table 1:** Advantages and disadvantages of various methods used in the preparation of peptides [29,30].

Preparation Method	Advantages	Disadvantages
Natural extraction	Simple operation	Natural raw materials, limited production, purification of natural peptides is difficult.
Chemical synthesis	Mainly used for the preparation of short peptides with short development cycle, rapid production, high purity	High cost, by-products, not environmentally friendly.
Chemical hydrolysis	The hydrolysis reaction is rapid, thorough, low cost, and low investment	Controlling hydrolytic peptides can be challenging due to the sensitivity of the amino acids, which are easily destroyed. Additionally, the resulting hydrolysate may have a dark appearance, and acid hydrolysis can produce the toxic substance chloropropanol.
Enzymatic hydrolysis	Mild conditions, high enzyme specificity, high product purity, and high hydrolysis efficiency	It is difficult to purify the peptide mixture by hydrolysis.
Genetic recombination	Mainly used for the preparation of larger peptides	The research and development process is challenging owing to the long development cycle and the immaturity of the related technology, with limited production ability of short peptides.
Fermentation	Waste resources can be used, reducing the economic burden	The selection of microorganisms is relatively strict and there are safety problems.

**Table 2:** Advantages and disadvantages of various methods for the separation and purification of peptides [37,38].

Separation and Purification of Peptides	Advantages	Disadvantages
Ultrafiltration	Peptides with specific molecular weight can be easily intercepted for industrial use	Poor separation ability
Reversed-phase high-performance liquid chromatography	Good separation effect and reproducibility	High cost
Ion-exchange chromatography	High resolution, large injection volume, acid and alkali resistance, and simple operation are appropriate for industrial scale-up of pure peptide processes	Consumables are expensive, slow, small range, and greatly affected by the environment
Gel filtration chromatography	The high resolution is conducive to the purification of small-molecular-weight peptides	Consumables are expensive
Hydrophilic interaction chromatography	High specificity	Carriers are expensive
Macroporous resin chromatographic column	Fast adsorption speed, gentle desorption conditions, easy regeneration treatment, long service period	The purity of the separation is relatively low

**Table 3:** Advantages and disadvantages of different methods for the identification of peptides [49,50].

Identification of Peptides	Advantages	Disadvantages
*N*-terminal sequencing	A traditional method for amino acid sequencing	Not effective for *N*-terminal blocked polypeptides
Nuclear magnetic resonance	Can be used to analyse the composition and amino acid sequence of each component in a quantitative mixture	Can only analyse small peptides with less than 30 amino acids
Mass spectrometry	High sensitivity, capability to detect *N*-terminally blocked peptides	High cost

**Table 4:** Research progress in the preparation, isolation, purification, identification, and activity of walnut neuroprotective peptides.

Raw Materials	Preparation	Isolation and Purification	Identification Method	In Vivo and In Vitro Models	Mechanism	Peptide Sequence	References
Defatted walnut meal	Hydrolysed at 55 °C, pH8.0 for 12 h by pancreatin (at a substrate to enzyme ratio of 20:1 *w*/*w*)	SP-825 macroporous adsorption resin; medium-pressure liquid chromatography	UPLC-ESI-MS/MS	H_2_O_2_-injured PC12 cells/D-galactose-induced learning and memory impairments in mice	(1) Antioxidant activity; (2) activating intracellular antioxidant enzymes (SOD and GSH-px) through Keap1 inhibition, inhibiting ROS production, Ca^2+^ influx, and MMP collapse as well as regulating the expression of apoptosis-related proteins	WSREEQEREE, ADIYTEEAGR	[39,105]
Defatted walnut meal	Hydrolysed at 55 °C, pH7.0 for 16 h with an enzyme mixture of pancreatin and viscozyme L (at a protease to substrate ratio of 0.8% *w*/*w*)	NA	NA	ORAC and ABTS assay/scopolamine-induced memory deficits in mice	(1) Regulating the cholinergic system (increasing the AChR amount and upregulating the mRNA expression of ChAT); (2) protecting neurons in the central nervous system from free radical damage (scavenging free radicals)	NA	[106]
Walnut protein	Hydrolysed by adding two proteases (complex plant hydrolase and pancreatin) at a protease/substrate ratio of 1.0% and 1.0% (*w*/*w*) in controlled conditions (pH 7.0, 55 °C for 12 h)	Ultrafiltration membrane (MW < 3 kDa), Sephadex G-15 gel filtration chromatography (2.6 × 70.0 cm^2^)	UPLC-ESI-QTOF-MS/MS	Memory deficits induced by sleep deprivation in rats/glutamate-induced apoptosis in PC12 cells	(1) Reduction of antioxidant defence (CAT, GSH-px, and SOD) and an increase of MDA content; (2) inhibiting Ca^2+^ influx and MMP collapse; (3) regulate the expression of apoptosis-related proteins (Bax and Bcl-2)	GGW, VYY, LLPF	[34]
Walnut protein	Viscozyme L (protease/substrate 1.0%, *w*/*w*) and pancreatin (protease/substrate 1.0%, *w*/*w*) at pH 7.0, 55 °C for 12 h	Ultrafiltration membrane (MW < 3 kDa), Sephadex G-15 gel filtration chromatography	UPLC–ESI-QTOF-MS/MS	LPS-activated inflammation in BV-2 cells/LPS-induced learning and memory deficits in mice	(1) Anti-inflammatory; (2) antioxidative properties	LPF, GVYY, APTLW	[89]
Walnut protein	Complex plant hydrolase and pancreatin with a 1.0% (*w*/*w*) enzyme/substrate ratio and hydrolysed at pH 7.0, 55 °C for 12 h	NA	UPLC-ESI-QTOF-MS/MS	Scopolamine-induced cognitive and memory impairment in mice and zebrafish	(1) Ameliorative effect on cholinergic system damage; (2) reducing oxidative stress	FY, SGFDAE	[36]
Defatted walnut meal	Simulated gastrointestinal digestion (pepsin/protein ratio of 1:10 *w*/*w* pH2.0, 37 °C, 3 h) and pancreatin (pancreatin/protein ratio of 1:10 *W*/*W*, pH7.4, 37 °C, 3 h) sequentially	Ultrafiltration, gel filtration chromatography, and RP-HPLC	UPLC-ESI-QTOF-MS	H_2_O_2_-stimulated SH-SY5Y cells/D-galactose and aluminium chloride administration to mice	(1) Alleviated oxidative stress; (2) reversed cholinergic dysfunction; (3) suppressed the release of proinflammatory cytokines in the brains of mice; (4) decline in the phosphorylation of JNK and P38 and the nuclear translation of Nrf2	TY, SGGY	[22,40,60]
Defatted walnut dregs	Dissociated with alkali protease (200 U/mg) over the course of 4 h at a substrate ratio (*E*/*S*) of 1:50 (*w*/*w*) at 55 °C.	Sephadex G-25	LC-ESI-MS/MS	HEK-293-E22G cell model of intracellular Aβ42 aggregation/APP/PS1 mouse model	(1) Reducing β-amyloid plaques in the brain; (2) alters the gut microbiota and serum metabolites compositions	PPKNW	[23,67]
Defatted walnut meal	Hydrolysed by compound proteases and alkaline proteaseat 50–55 °C with agitation for 18–24 h	Ultracentrifugation (8000 rpm, 20 min)	HPLC-MALDI-TOF-MS, de novo sequencing	H_2_O_2_-injured SH-SY5Y cells/scopolamine-induced learning and memory deficits in mice	(1) Hydroxyl radical scavenging; (2) ROS reduction	VEGNLQVLRPR, LAGNPHQQQQN, HNLDTQTESDV, AGNDGFEYVTLK, AELQVVDHLGQTV, EQEEEESTGRMK, QQRQQQGI, WSVWEQELEDR	[48,83]
Manchurian walnuts	Fermented for 3 h separately using neutrase (9000 U/g) at pH 7.0 and 52.5 °C and using alcalase (7000 U/g) at pH 8.4 and 55.5 °C	Ultrafiltration (>10 kDa, 3–10 kDa, <3 kDa)		H_2_O_2_-induced PC12 cells/ scopolamine-induced in mice	(1) Reduction of oxidative stress; (2) inhibition of neural cell apoptosis; (3) regulation of various neurotransmitters; (4) maintaining hippocampal CA3 pyramidal neurons and upregulation of p-CaMK II levels	Manchurian walnut hydrolysed peptide (<3 kDa)	[107]
Manchurian walnuts	NA	Sephadex G-15, RP-HPLC	HPLC-ESI-Q-TOF-MS/MS	H_2_O_2_-induced PC12 cells	(1) Reducing ROS generation and enhancing intracellular antioxidant enzymes (SOD, CAT and GSH-px); (2) suppressed the expression of IKKβ and p65 to inhibit NF-κB pathway activation, attenuating the neurotoxic cascade by overexpression of IL-1β and TNF-α; (3) inhibited apoptosis by suppressing the caspase signal pathway; (4) upregulated the expression of p-CREB and synaptophysin	EVSGPGLSPN	[90]
Walnut-derived peptide	Chemical synthesis	NA	NA	H_2_O_2_-treated HT-22 cells/scopolamine-induced cognitive-impaired mice	(1) Alleviating oxidative stress; (2) promoted the expression of mitophagy-related proteins and activated the NRF2/KEAP1/HO-1 pathway	TWLPLPR, YVLLPSPK, KVPPLLY	[56]
Walnut-derived peptide	Chemical synthesis	NA	NA	LPS-stimulated BV-2 microglia	(1) Reducing ROS generation and enhancing antioxidant enzymes (SOD and CAT) activity; (2) reducing NO generation, attenuating inflammatory factors (TNF-α, IL-1β, IL-6), and decreasing the expression of inflammatory response-related enzymes (iNOS and COX2); (3) activating the Nrf2/HO-1 pathway and inhibiting the NF-κB/p38 MAPK pathway	WEKPPVSH	[58]
Walnut-derived peptide	Chemical synthesis	NA	NA	Scopolamine-injured mice	Maintains lysosome homeostasis	EVSGPGLSPN	[69]
Walnut-derived peptide	Chemical synthesis	NA	NA	D-galactose-induced mice/Aβ_25–35_–injured bend.3 cells	Maintains the blood-brain barrier integrity by inhibiting the expression and activity of matrix metalloproteinase 9	TWLPLPR	[65]
Walnut-derived peptide	Enzyme hydrolysis, chemical synthesis	NA	NA	Scopolamine-induced cognitive deficits in mice/LPS-induced THP-1 cells	Decreased the activities of DNA methyltransferases	Walnut hydrolysate proteins (<3 kDa) YVLLPSPK	[108]
Walnut protein	Alkaline protease (pH 9.0, 55 °C) hydrolysis	Ultrafiltration	LC-MS/MS	D-galactose-induced cognitive-impaired mice	(1) Inhibiting oxidative stress; (2) inhibiting neuroinflammation; (3) modulating the gut microbiota and serum metabolite compositions.	RLWPF, VLRLF	[33]

NA, information not available; UPLC-ESI-MS/MS, ultra-high performance liquid chromatography-electrospray ionisation-tandem mass spectrometry; MALDI, matrix-assisted laser desorption/ionisation; MW, molecular weight; QTOF, quadrupole time of flight; RP, reversed phase; SOD, superoxide dismutase; GSH-px, glutathione peroxidase; ROS, reactive oxygen species; ORAC, oxygen radical absorbance capacity; CAT, catalase; MDA, malondialdehyde; Keap1, Kelch-like ECH-associated protein 1; MMP, mitochondrial membrane potential; TNF-α, tumour necrosis factor-alpha; IL-1β, interleukin-1β; IL-6, interleukin-6; iNOS, nitric oxide synthase; COX2, cyclooxygenase-2; NF-κB/p38 MAPK, nuclear factor-κB/p38 mitogen-activated protein kinase; Nrf2/HO-1, nuclear factor erythroid 2-related factor 2/heme oxygenase-1.

## Data Availability

Not applicable.
